# Obesity-associated insulin resistance is correlated to adipose tissue vascular endothelial growth factors and metalloproteinase levels

**DOI:** 10.1186/1472-6793-12-4

**Published:** 2012-04-02

**Authors:** Francisco José Tinahones, Leticia Coín-Aragüez, Maria Dolores Mayas, Eduardo Garcia-Fuentes, Carmen Hurtado-del-Pozo, Joan Vendrell, Fernando Cardona, Rosa-Maria Calvo, Maria-Jesus Obregon, Rajaa El Bekay

**Affiliations:** 1 CIBER Fisiopatología Obesidad y Nutrición (CB06/03), Instituto de Salud Carlos III, Madrid, Spain; 2 Laboratorio de Investigación Biomédica, Hospital Clínico Universitario Virgen de la Victoria, Campus de Teatinos s/n, 29010 Málaga, Spain; 3 Servicio de Endocrinología, Hospital Virgen de la Victoria, Malaga, Spain; 4 Instituto de Investigaciones Biomedicas (IIBM, CSIC-UAM]), Madrid, Spain; 5 CIBERDEM. University Hospital of Tarragona Joan XXIII. IISPV, Rovira i Virgili University, Tarragona, Spain

**Keywords:** Vascular Endothelial Growth Factor and Metalloproteinase, Obesity, Insulin Resistance, Omentum Adipose Tissue, Subcutaneous Adipose Tissue

## Abstract

**Background:**

The expansion of adipose tissue is linked to the development of its vasculature, which appears to have the potential to regulate the onset of obesity. However, at present, there are no studies highlighting the relationship between human adipose tissue angiogenesis and obesity-associated insulin resistance (IR).

**Results:**

Our aim was to analyze and compare angiogenic factor expression levels in both subcutaneous (SC) and omentum (OM) adipose tissues from morbidly obese patients (n = 26) with low (OB/L-IR) (healthy obese) and high (OB/H-IR) degrees of IR, and lean controls (n = 17). Another objective was to examine angiogenic factor correlations with obesity and IR.

Here we found that *VEGF-A *was the isoform with higher expression in both OM and SC adipose tissues, and was up-regulated 3-fold, together with *MMP9 *in OB/L-IR as compared to leans. This up-regulation decreased by 23% in OB/-H-IR compared to OB/L-IR. On the contrary, *VEGF-B*, *VEGF-C *and *VEGF-D*, together with *MMP15 *was down-regulated in both OB/H-IR and OB/L-IR compared to lean patients. Moreover, *MMP9 *correlated positively and *VEGF-C*, *VEGF-D *and *MMP15 *correlated negatively with HOMA-IR, in both SC and OM.

**Conclusion:**

We hereby propose that the alteration in *MMP15*, *VEGF-B*, *VEGF-C *and *VEGF-D *gene expression may be caused by one of the relevant adipose tissue processes related to the development of IR, and the up-regulation of *VEGF-A *in adipose tissue could have a relationship with the prevention of this pathology.

## Background

The prevalence of obesity has increased dramatically in the last decades and is now considered a major health problem. In fact, the current epidemic of obesity has been suggested as the leading cause for the decreased life expectancy forecast for the next generation. Obesity is very often accompanied by other diseases, the most common being type 2 diabetes mellitus (T2DM) and cardiovascular complications [1-3].

T2DM and obesity both involve genetic and environmental factors. However, progression to overt diabetes in patients with obesity is not clearly predicted. Likewise, while some obese individuals progress to T2DM, others only have mild metabolic abnormalities suggesting that the absolute amount of fat stored may not be the most important factor in determining the relationship between obesity and T2DM [4-7].

Adipose tissue expandability in response to positive energy balance has traditionally been considered an adaptive passive process. However, recent evidence suggests that the expandability of adipose tissue is not an unlimited process. In fact, adipose tissue expandability may be an important factor determining the appearance of obesity-associated co-morbidities [7-9]. Several studies have shown the prevalence of a negative IR status and no complications in patients with severe morbid obesity (Metabolically healthy, but obese individuals) [10-12].

Adipose tissue expansion is well known to be linked to the development of its vasculature [13]. Furthermore, it has been described that obesity is associated with extensive modifications in adipose tissue involving adipogenesis, angiogenesis and proteolysis [14]. However, until now, no studies have established the relationship between adipose tissue angiogenic capacity, obesity and IR.

It is generally well known that the vascular endothelial growth factor (VEGF) system accounts for most of the angiogenic activity in adipose tissue [15]. *VEGF-A *(17-23 kDa) is a major angiogenic factor that stimulates proliferation and migration of ECs [16]. *VEGF-B *(21 kDa) is 43% identical to *VEGF-A*; it also promotes angiogenesis and is implicated in extracellular matrix (ECM) degradation via the regulation of plasminogen activation [17]. *VEGF-C *displays a 30% homology with *VEGF-A*, and plays an important role in both angiogenesis and lymphangiogenesis [18,19]. *VEGF-D *is 48% identical to *VEGF-C *and also promotes the growth of lymphatic vessels [20]. Matrix metalloproteinases (MMPs) are essential for proper ECM remodeling, a process that takes place during obesity-mediated adipose tissue formation. The development of obesity is associated with coordinated cellular processes, including adipocyte hypertrophy followed by recruitment of adipocyte precursors, and new fat cell differentiation [21,22]. These processes are also accompanied by neovascularization, essential for the generation and proper function of adipose tissue [23]. It is generally accepted that such multiple events include dynamic changes of cell-matrix interactions and extensive ECM remodeling, and that modifications in proteolytic activities within the adipose microenvironment might occur during the development of the fat depot. Among enzymes implicated in the degradation of matrix molecules and in the generation of bioactive factors, the matrix metalloproteinase family is considered to be primarily responsible for these processes [24]. These subgroups are *collagenases*, *gelatinases*, *stromelysins*, *membrane-type MMPs *(*MT-MMPs*), and other MMPs [25,26]. MMPs participate in many physiological and pathological processes such as embryonic development, angiogenesis, wound repair, reproductive cycling, and metastasis [24,25]. Also, they can mediate the release and/or activation of sequestered growth factors, including *VEGFs*, and the cleavage of cell surface adhesion receptors [25].

On the basis of all these antecedents our aim was to analyze angiogenic factor and metalloproteinases expression levels in adipose tissue from control and obese subjects with or without IR, to study the relationship between adipose tissue-angiogenic factors/metalloproteinase, obesity and IR.

## Results

### Anthropometric and biochemical characteristics of the patients

The anthropometric and biochemical parameters from controls and obese subjects (with low and with high insulin resistance) are depicted in Table 1. The severely obese patient group had a significantly higher body mass index (BMI) and waist circumference (WC) (57.27 ± 2.07 kg/m2 and 144.4 ± 7.14) (*p *< 0.05 and *p *< 0.01 respectively) with respect to lean subjects (Controls). No anthropometric differences were observed between the two groups of obese subjects with low and high degrees of insulin resistance. Table 1 also illustrates the biochemical abnormalities associated to obesity and IR, showing low levels of HDL cholesterol in morbidly obese patients as compared to lean subjects. Adiponectin, a protein hormone produced and secreted by adipocytes and known to regulate the metabolism of lipids and glucose and related to angiogenesis in adipose tissue showed significantly low levels in obese patients (*p *< 0.01). There were no differences in the homeostasis model assessment (HOMA) indices between healthy OB/L-IR and controls, and both groups in turn displayed significant differences in this index respect to the OB/H-IR group.

**Table 1:** Clinicopathological characteristics and biomarker parameters

	Control (n = 17)	OB/non IR (n = 12)	OB/H-IR (n = 14)	*p*
**Age (years)**	44.46 ± 2.2^a^	43.64 ± 3.22^a^	37.50 ± 2.90^a^	-

**BMI**	23.04 ± 0.32^a^	57.28 ± 2.40^b^	57.26 ± 1.75^b^	< 0.05

**Triglycerides (mM)**	1.14 ± 0.15	1.19 ± 0.15	1.13 ± 0.36	-

**HOMAindex**	1.23 ± 0.14^a^	3.31 ± 0.24^a^	13.79 ± 1.28^b^	< 0.01

**Adiponectin (μg/mL)**	12.48 ± 1.26^a^	10.83 ± 1.54^b^	9.07 ± 1.52^b^	< 0.01

**Cholesterol (mM)**	4.91 ± 0.29^a^	5.38 ± 0.45^a^	5.02 ± 0.19^a^	-

**Cholesterol HDL (mM)**	1.42 ± 0.14a	0.93 ± 0.20^b^	1.04 ± 0.12^b^	0.05

**Waist circumference**	82.03 ± 1.95	142.8 ± 6.88	146.0 ± 7.41	< 0.01

Data are mean ± SEM. *BMI*: body mass index, *HOMA *index: homeostasis model assessment index. Comparison of high degree insulin resistant (*OB/H-IR*) and low degree insulin resistant (*OB/L-IR*) obese subjects versus controls was assessed by ANOVA test. Groups with different letters showed significant differences.

### Angiogenesis related gene expression profiles in OM and SC adipose tissues from leans, OB/H-IR and OB/L-IR subjects

**Figure 1:**
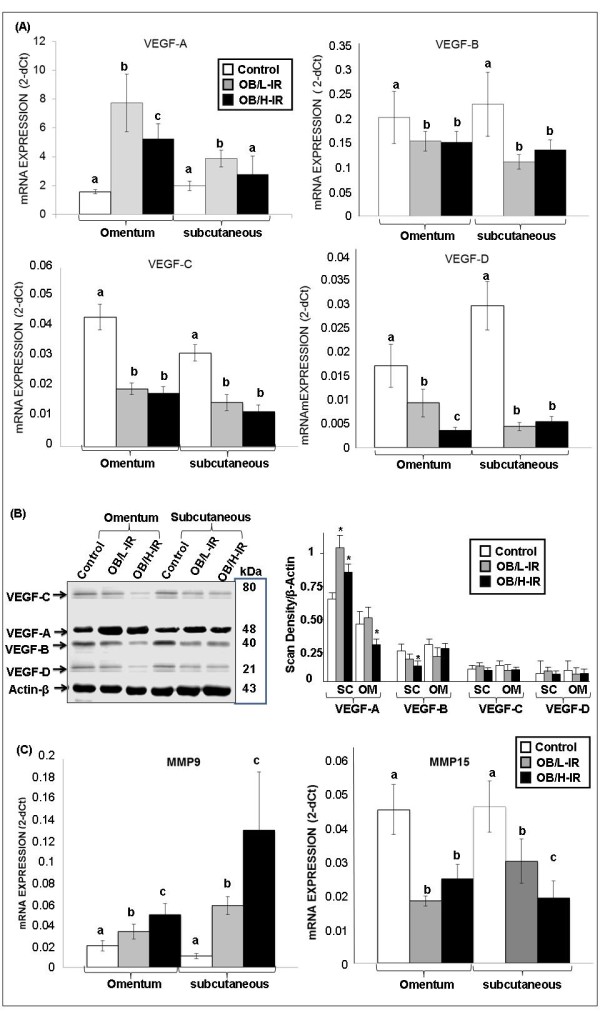
***VEGF *isoforms, *MMP9*, *MMP15 *mRNA and protein expression in omentum and subcutaneous AT from subjects**. (A) *VEGF *isoforms [*VEGF-A*, *VEGF-B*, *VEGF-C *and *VEGF-D*] mRNA expression analysis was performed on human omentum (OM) and subcutaneous (SC) adipose tissues from three subject groups (lean, OB/H-IR and OB/L-IR subjects). mRNAs were normalized to *cyclophilin *levels. Results were obtained from triplicate and expressed as the mean ± SEM (n = 17 for controls, n = 12 for OB/L-IR and n = 14 for OB/H-IR). Bars with different letters have a significant difference, *p *< 0.05. (B) Immunoblotting analysis of *VEGF *isoforms. The resulted blot of each isoform was compared to *Actin-β *constitutive protein. Density analyze was done normalizing the samples to β-actin using NIH's Image-J software program. The blot is representative of three independent experiments with different samples, * *p *< 0.05. (C) Metalloproteinase (*MMP9 *and *MMP15*) mRNA expression analysis was performed on human omentum (OM) and subcutaneous (SC) adipose tissues from three groups of subjects (leans, OB/H-IR and OB/L-IR). mRNAs were normalized to *cyclophilin *levels. Bars with different letters have a significant difference (*p *< 0.05); differences are compared to the control and between the two groups of obese.

We performed mRNA expression analysis in a set of genes involved in the regulation of angiogenesis in OM and SC samples from lean and obese subjects both with low and high degrees of IR. These genes are basically *VEGF *isoforms and proteolytic MMPs. Figure 1A shows that *VEGF-A *gene expression was significantly enhanced in OM from OB/L-IR (by about 3 fold) as compared to lean subjects. *VEGF-A *gene expression up-regulation was significantly reduced by about 23% in OB/H-IR subjects as compared to OB/L-IR. In SC, *VEGF-A *levels were lower than those observed in OM, however the up-regulation of this angiogenic form was also observed in both OB/L-IR and OB/H-IR subjects compared to leans. These data indicate the high up-regulation of *VEGF-A *isoform in both SC and OM adipose tissues from healthy morbidly obese subjects and this up-regulation was diminished in morbidly obese subjects with IR.

However, *VEGF-B *gene expression was significantly down-regulated in both OM and SC from OB/H-IR and OB/L-IR subjects compared to leans, and no significant differences were observed between OB/H-IR and OB/L-IR subjects. Both *VEGF- C *and *VEGF-D *gene expressions showed a drastic and significant down-regulation in both OM and SC from OB/H-IR and OB/L-IR subjects compared to leans. Moreover, *VEGF- B*, *VEGF-C *and *VEGF-D *expression levels were much lower in both OM and SC as compared to *VEGF-A *isoforms.

*VEGF *isoform protein expressions were also analyzed in both OM and SC from leans and OB/H-IR and OB/L-IR subjects. Figure 1B shows evidence for the presence of protein synthesis of the four *VEGF *isoforms in both OM and SC in all the subjects. These protein analyses confirmed the results obtained by real time PCR, showing that *VEGF-A *is the isoform more abundant in both OM and SC as compared to the other isoforms, and that this isoform is especially enhanced in obese subjects compared to leans. In addition, we observed that *VEGF-B*, *VEGF-C *and *VEGF-D *protein expression was decreased in obese subjects compared to leans, especially in those with high degrees of insulin resistance (Figure 1B).

*MMP9 *gene expression levels were significantly enhanced especially in SC from both OB/H-IR and OB/L-IR subjects compared with leans (Figure 1C). In fact, in OM, *MMP9 *expression was about 25% higher in OB/L-IR subjects than in leans, and about 50% higher in OB/H-IR compared to leans. In SC, the differences in *MMP9 *gene expression were even higher than in OM. In fact, SC *MMP9 *gene expression was 3-fold higher in OB/L-IR subjects than in leans, and about 6-fold higher in OB/H-IR subjects than in leans. When comparing SC *MMP9 *gene expression in the two obese groups, its expression was 80% higher in OB/H-IR subjects than in OB/L-IR (Figure 1C). Opposite, *MMP15 *expression showed significant down-regulation in both OM and SC from both OB/H-IR and OB/L-IR subjects compared to leans (Figure 1C). In fact, OM *MMP15 *gene expression was lower by approximately 50% in OB/H-IR and OB/L-IR subjects compared to leans, and this difference was statistically significant. Also, SC *MMP15 *gene expression levels were approximately 33% lower in OB/L-IR subjects than in leans and by about 40% lower in OB/H-IR compared to leans.

### Correlation analysis with HOMA and IR

**Figure 2:**
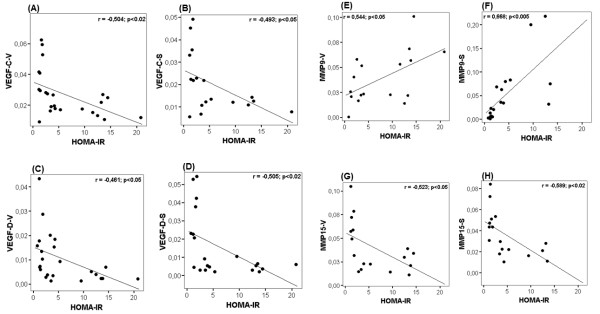
**Correlative analysis of *VEGF *isoforms and *MMPs *with the HOMA index**. Correlation of *VEGF-C *and *VEGF-D *isoforms, *MMP15 *and *MMP9 *mRNA expression with the HOMA index from both omentum (*OM*) and subcutaneous (*SC*) adipose tissues was determined by Pearson's correlation coefficient test (*r*).

Correlation analysis of the different genes studied with HOMA, the parameter determining the subjects' insulin resistance indicated that *VEGF-C *and *VEGF-D *from both SC and OM correlated negatively with the HOMA index (Figure 2A-D). Nevertheless, when we examined the correlation between *VEGF-A *and HOMA, no correlations was observed in both OM and SC. Moreover, we observed that *MMP9 *metalloproteinase correlated positively with HOMA (Figure 2E and 2F). However, *MMP15 *showed a clear negative correlation with HOMA in both SC and OM tissues (Figure 2G and 2H). On the other hand, when we analyzed the correlations between *VEGF *isoforms and the *MMPs *we noted that *MMP15 *correlates positively with *VEGF-C *(r = 0.53, *p *< 0.02). *MMP9 *showed a significant negative correlation with *VEGF-C *(r = -0.48, *p *< 0.05), and positive correlation with *VEGF-A *(r = 0.33, *p *< 0.05). Moreover, analyzing the potential correlations between *VEGF *isoforms, we observed a positive and significant correlation between *VEGF-C *and *VEGF-B *(r = 0.60, *p *< 0.05) and between *VEGF-C *and *VEGF-D *(r = 0.77, *p *< 0.001). However, no significant correlation was observed between *VEGF-A *and others *VEGF *isoforms. On the other hand, when we analyzed the correlations between these angiogenic parameters and HOMA index in obese group, we observed that the results showed the same trends as in above statistical analysis, although some correlations did not show significant differences (Table 2)

**Table 2:** Correlations between VEGF isoforms and MMPs gene expression with HOMA-IR in adipose tissue from obese subjects

	Rs	*p*
**VEGF-C-V/HOMA IR**	-0.404	0.054

**VEGF-C-S/HOMA IR**	-0.412*	0.036

**VEGF-D-V/HOMA IR**	-0.403*	0.021

**MMP9-V/HOMA IR**	0.439*	0.041

**MMP9-S/HOMA IR**	0.417	0.052

**MMP15-S/HOMA IR**	-0.357	0.0652

Correlations were determined by Pearson's correlation coefficient test *p < 0.05.

## Discussion

Modulation of angiogenesis can potentially impair the development of obesity-associated co-morbidities. However, little is known of the relationship between angiogenic components and insulin resistance in human adipose tissues.

The fact that some morbidly obese subjects having VEGF-A overexpressed in their adipose tissue did not develop IR leads us to believe that the enhancement of VEGF-A, which is known be responsible for most of adipose tissue's angiogenic capacity [26], could probably be in response to the impaired lymphangiogenic capacity, which was reflected by VEGF-B, VEGF-C and VEGF-D reduction. These mechanisms may somehow have some relationship in preventing IR. In line with our findings, it has been recently described that during differentiation of 3 T3-F442A preadipocytes, VEGF-B and VEGF-C mRNA levels are less modulated, whereas that of VEGF-A are up-regulated [27]. Moreover, other data show that in patients with abdominal obesity, stimulation of SAT angiogenesis associated to insulin resistance promotes SAT hyperplasia, thus enhancing the storage capability of adipose tissue [28]. Our suggestions may appear speculative, but we consider that the significant negative correlation of VEGF-C and VEGF-D with the HOMA index points to the possible relationship between down regulation of these two isoforms and insulin resistance development in morbidly obese subjects. Our hypothesis is that down-regulation of these angiogenic factors could be related to an alteration in the insulin sensitivity signaling pathway, as it is well known that insulin sensitivity signaling pathways involve a variety of genes including VEGF, GLUT1 and hypoxia-inducible factor HIF-1α/ARNT [29].

MMPs are proteolytic enzymes that play an essential role in extracellular matrix remodeling and VEGF regulation. MMPs are involved in two important events of this process and control proteolysis and adipogenesis during obesity-mediated fat mass development [30,31]. Several previous studies have suggested a role for MMPs in adipose tissue remodeling. Other studies have reported altered levels of MMPs in disorders related to insulin resistance [32]. Here, the up-regulation of gene expression of MMP9 and its positive correlation with the HOMA index point to the potential relationship between this metalloproteinase and insulin resistance development in morbidly obese subjects. In accordance with our suggestions, recent studies showed that MMP-9 expression in adipose tissue is increased with obesity and insulin resistance and is increased in adipocytes, and this increase in MMP-9 was related to the regulation of mediators of insulin signaling pathways such as PKCα and PPARγ [33]. Other researchers have provided the first evidence that human adipose tissue releases MMP-9 and that this secretion is modulated during adipocyte differentiation [34]. Recent studies have also shown that MMP9 expression was elevated in preadipocytes and the stroma vascular fraction of obese subjects compared with those of non-obese subjects [35].

We can say that we are the first to evaluate MMP-15 in adipose tissue and to propose the potential relationship between it, obesity and insulin resistance. Down regulation of gene expression MMP-15 and its significant negative correlation with the HOMA index point to the existence of a potential relationship between this metalloproteinase, VEGF-C, VEGF-D and insulin resistance in morbidly obese subjects.

## Conclusions

In summary, our study highlights a group composed by the angiogenic factor VEGF-A and a relevant regulator of ECM degradation, MMP9, which in our opinion, could be acting together in adipose tissue as a compensatory mechanism that replaces the alteration in VEGF-B, VEGF-C, VEGF-D and MMP15. These data add another fact related to the metabolic derangements of obesity, including IR to the disturbance of one of the functionalities of adipose tissue controlled by VEGF and metalloproteinase angiogenic factors factors.

Finally, we believe that a better understanding of the regulation of the expression of these angiogenic components in adipose tissue will thus be instrumental in the development of specific therapeutic targeting approaches.

## Methods

### Patients and adipose tissue collection

The study was performed in morbidly obese patients (n = 26) undergoing bariatric surgery at the Hospital Clínico Virgen de la Victoria. Exclusion criteria were: T2DM treated with insulin, cardiovascular disease in the 6 months prior to inclusion in the study, evidence of acute or chronic inflammatory disease, infectious disease or the patient's decision not to participate in the study.

Control subjects (n = 17) were patients who underwent laparoscopic surgery for hiatus hernia or cholelithiasis, who were not obese, with a similar age to the morbidly obese group, and with the same selection criteria.

The study groups were organized by:

- Morbidly obese: High insulin resistance (HOMA IR > 9) shown in the text as (OB/H-IR). Low insulin resistance (HOMA IR < 4) shown in the text as (OB/L-IR). The cut-off was set at average plus two standard deviations of the values of a healthy control population.

- Controls: BMI 18.5-24.9 without insulin resistance

All participants gave their informed consent, and the study was reviewed and approved by the ethics and research committee of the Vírgen de la Victoria Clinical University Hospital (Malaga, Spain).

Both SC and OM adipose tissues were obtained at the beginning of the surgical procedure and were stored immediately at -80°C.

### RNA extraction

Total RNA was isolated from adipose tissue using the Trizol RNA isolation method (Invitrogen, Carlsbad, CA) and purified with the RNeasy Lipid kit (QIAGEN -Ref. 74804-, Valencia, CA).

### Real-time PCR

Amplifications were performed using a MicroAmp^® ^Optical 96-well reaction plate (PE Applied Biosystems) on an ABI 7500 Real-Time PCR System (Applied Biosystems). RT qPCR reactions were carried out for all genes using specific TaqMan^® ^Gene Expression Assays. During PCR, the Ct values for each amplified product were determined using a threshold value of 0.1. The specific signals were normalized by constitutively expressed *cyclophilin *(*Cyc: *Ref. 4326316E) signals using the formula 2^-ΔCt^.

### TaqMan^® ^gene expression assay probes

*VEGF-A*: Ref. Hs00173626_m1. Sequence: NM_001025366.2

*VEGF-B*: Ref. Hs00957984_m1. Sequence: NM_003377.3

*VEGF-C*: Ref. Hs00153458_m1. Sequence: NM_005429.2

*VEGF-D*: Ref. Hs01128659_m1. Sequence: NM_004469.4

*MMP9*: Ref. Hs00234579_m1. Sequence: NM_004994.2

*MMP15*: Ref. Hs00233997_m1. Sequence: NM_002428.2

### Western blot analysis

Total proteins from adipose tissues were extracted by *NE-PER Nuclear and Cytoplasmatic Extraction Reagents protocol *(Pierce -Ref.78833-). Protein extracts (30 μg) were separated by SDS-PAGE, blotted onto a PVDF membrane and then incubated with specific antibodies (*VEGF (A-20): *sc-152; *VEGF-B (J-14I): *sc-80442; *VEGF-C (H-48): *sc-25783; *VEGF-D (X142): *sc-80447; Santa Cruz Biotechnology, INC. *Monoclonal Anti-B-Actin: *A5316; Sigma Aldrich). Protein signals were detected by electrochemiluminescence detection Quantity One^® ^software (Bio-Rad Laboratories).

### Statistical analysis

SPSS Inc. software (Version 15.0) was used for all statistical analyses. Comparisons between the normalized mRNA levels of different genes and the biochemical characteristics of the groups used in this study were made by ANOVA test and the statistical differences between the groups were made by Duncan's test. The correlation analysis of mRNA quantitative expression of each of the genes was performed with Pearson's coefficient Test (*r*).

## Competing interests

The authors declare that they have no competing interests.

## Authors' contributions

FJT financed part of the study, participated in the design of the study and helped to draft the manuscript, LCA and MDM carried out the molecular biology studies, EGF helped coordinate and collect samples from patients, CHDP, RMC, JV and FC participated in the molecular biology studies, and MJO participated in the molecular biology studies and financed part of the study. REB conceived, designed and coordinated the study, performed the statistical analysis and drafted the manuscript. All authors read and approved the final manuscript.
